# Differences in sensory-motor cortex activation patterns during level and stair walking in patients with knee osteoarthritis: protocol for a cross-sectional study

**DOI:** 10.3389/fnagi.2025.1589645

**Published:** 2025-05-19

**Authors:** Qian Deng, Guohua Jin, Xia Lou, Yuwu Ding, Haichen Xu, Kun Yang, Bingli Liu, Liming Jiang

**Affiliations:** ^1^School of Health and Nursing, Wuxi Taihu University, Wuxi, China; ^2^Department of Rehabilitation, Seventh People’s Hospital of Shanghai University of Traditional Chinese Medicine, Shanghai, China; ^3^Department of Orthopedic Ward II, Zhuji Hospital of Traditional Chinese Medicine, Zhejiang, China; ^4^Department of Orthopedics and Traumatology, Seventh People’s Hospital of Shanghai University of Traditional Chinese Medicine, Shanghai, China

**Keywords:** knee osteoarthritis, cortex activation, functional near-infrared spectroscopy, level walking, stair walking, surface electromyography

## Abstract

**Background:**

Knee osteoarthritis (KOA) is a chronic degenerative disease characterized primarily by pain and joint dysfunction, especially during level and stair walking. Although traditionally classified as a peripheral joint disease, emerging evidence implicates central nervous system (CNS) abnormality in KOA pathogenesis. Our previous studies found that KOA patients showed decreased activation in sensory-motor cortex during isolated joint movements. However, it is not yet clear how brain activation patterns change during level and stair walking. Therefore, this study will investigate the sensory-motor cortex activation in KOA patients during different walking environments, providing evidence for potential targets for KOA central interventions.

**Methods:**

This study is designed as a cross-sectional observation, aiming to recruit 20 KOA patients and 20 demographically similar healthy controls (HC). Functional near-infrared spectroscopy (fNIRS) is utilized to assess the hemodynamic responses in the cerebral cortex within the specified regions of interest (ROIs), including the primary somatosensory cortex (S1), primary motor cortex (M1), and somatosensory association cortex (SAC). These measurements will be taken during three motor tasks: level walking, ascending stairs, and descending stairs. Simultaneously, surface electromyography (sEMG) is employed to measure muscle activity of the key muscle groups around the knee joint. The VAS and the WOMAC used to evaluate pain and functional symptoms in KOA patients, respectively. Subsequently, the potential correlations between cerebral hemodynamics changes within ROIs and clinical indicators are analyzed.

**Discussion:**

This study, based on “differential activation of the sensory-motor cortex under movement,” innovatively observes the relationship between pain and functional impairment in KOA patients and activation levels in specific brain regions across different motor environments. This not only provides a basis for early prediction of KOA onset but also offers potential targets for clinical interventions in KOA. Ultimately, the results of this study may open new perspectives for the rehabilitation of chronic musculoskeletal diseases.

## 1 Introduction

Knee osteoarthritis (KOA) is a common chronic joint disease characterized by pain and functional limitations (Jang et al., 2021; Katz et al., 2021; Alves-Simões, 2022). Epidemiological studies indicate that the incidence of knee osteoarthritis significantly increases with age, particularly among the elderly over 65, where the prevalence rate can reach 50%–60% (Bryk and Starowicz, 2021). With disease progression, it not only severely reduces the quality of life for patients but also exerts a heavy economic burden on society (Wood et al., 2013; Cohen et al., 2021). Despite the fact that there are currently multiple treatment approaches for KOA, including pharmacological treatment, physical therapy and surgical intervention, these methods frequently have limitations such as drug-induced toxic effects on other organ systems (e.g., gastrointestinal, cardiovascular, and renal systems), variable efficacy, and long-term financial burdens associated with chronic medication use or postoperative rehabilitation (Michael et al., 2010; Mahmoudian et al., 2021; Gelber, 2024). Hence, in-depth studies on the pathogenesis of KOA and its related factors (e.g., neurological factors) are particularly crucial, which will facilitate our development of more effective treatment strategies (Primorac et al., 2020; Du et al., 2023).

At present, studies for KOA mainly concentrates on the clinical characteristics and pathophysiological changes, but relatively less attention is placed on changes in the central nervous system (Dell’Isola et al., 2016; Yang et al., 2023). With the advancement of research, researchers have gradually realized that KOA patients had changes in brain structure and function, and these changes are closely related to pain perception and dysfunction (Shanahan et al., 2015; Liu et al., 2019; Barroso et al., 2020; Kang et al., 2021; Cheng et al., 2022). Studies have demonstrated that significant changes have occurred in the brain morphology of patients with KOA, including reductions in gray matter volume and changes in white matter integrity and static activity (Kang et al., 2021; Cheng et al., 2022). A magnetic resonance imaging (MRI) study using voxel-based morphometry (a neuroimaging technique for detecting structural brain changes) discovered that KOA patients had a decreased gray matter in regions related to pain processing and emotion regulation, such as the anterior cingulate cortex and insula (Kang et al., 2021). These changes might be associated with the persistence of chronic pain and modifications in the mechanisms of pain perception and regulation. The same study using MRI found that significant alterations occurred in the activation sites of the motor cortex in patients with KOA during the execution of isometric motion, which were linked to modifications in motor functions and behaviors (Shanahan et al., 2015). The authors suggest that these changes could be a significant contributing factor to the impaired motor performance observed in patients with KOA. Furthermore, another study using electroencephalogram (EEG) revealed distinct event-related desynchronization (decreased cortical oscillations reflecting neural activation) and synchronization (increased rhythmic activity associated with inhibitory processes) patterns in the EEG of KOA patients performing motor imagery tasks compared to healthy individuals (Marques et al., 2023). These findings imply that the brain function of KOA patients may be affected by chronic pain and functional limitations, leading to decreased motor control and sensory function. Nevertheless, existing research focusing solely on the resting state have failed to capture the brain activation patterns associated with dynamic physical movement, which are essential for a comprehensive perspective on KOA.

During movement execution, joints are subjected to greater loads and pressures, requiring a higher degree of sensorimotor integration, which may trigger more pronounced pain perception and changes in the activation of related functional brain regions (Marriott et al., 2019; Hutchison et al., 2022; Pan et al., 2024). Investigating these activation changes not only enhances our understanding of the neural mechanisms of pain processing, but also reveal possible impairments in patients’ motor performance. In order to study the activation changes in brain regions under dynamic knee movements, our research group previously employed functional near-infrared spectroscopy (fNIRS) to observe the activation of sensory-motor related brain regions in KOA patients during isokinetic knee movement and ultimately found that these brain regions were in a state of low activation (Yang et al., 2024). Nevertheless, the aforementioned study was limited to observations in specific environments, which differed significantly from the real-life daily activity environment. Bishnoi’s investigation focused on understanding how confronting unexpected challenges during gait—specifically, perturbation walking—affects prefrontal cortical activation in older women diagnosed with osteoarthritis (Bishnoi et al., 2024). The study found that prefrontal cortex activation increased significantly during perturbation walking compared to comfort walking, and this increase was more significant in healthy elderly women. However, the author only observed activation of the prefrontal cortex, and this observation was limited to the treadmill, which was different from daily walking. To clarify the above circumstances, our research group intends to further understand the activation patterns of the sensorimotor cortex of KOA patients in a real walking environment, including level walking and stair walking. Dynamic loading during gait and stair walking, characterized by high volume and frequency, is a fundamental aspect of human daily activities (Andriacchi et al., 2009). Notably, functional mobility limitations are frequently reported as primary challenging aspects of their condition among patients with KOA (Katz et al., 2021).

This study adopts a cross-sectional research design and utilized fNIRS to investigate brain activities changes in KOA patients under different movement contexts. The specific objective is to analyze the correlation between these changes and the patients’ pain and functional scores. This will provide a more powerful theoretical basis for the central intervention programs for KOA patients.

## 2 Materials and methods

### 2.1 Study design

**FIGURE 1:**
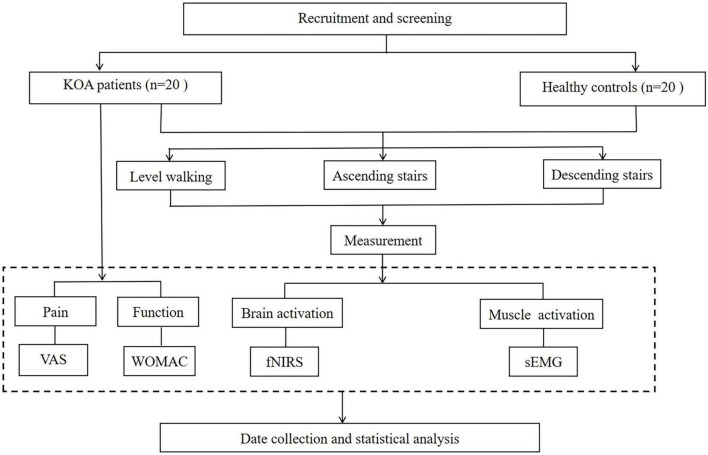
A brief flowchart of the entire study. KOA, knee osteoarthritis; VAS, visual analog scale; WOMAC, Western Ontario and McMaster Universities Osteoarthritis Index; fNIRS, functional near-infrared spectroscopy; sEMG, surface electromyography.

This study will adopt a cross-sectional study design comparing the differences in brain activation patterns and lower limb muscle activation patterns between KOA patients and Healthy controls (HC) during level and stair walking, and investigating their relationship with pain degree and dysfunction in KOA patients. A brief flowchart of the entire study is shown in Figure 1. The study protocol’s adherence to the STROBE checklist for cross-sectional studies signifies that all aspects of study design, execution, and reporting will follow established best practices for observational research (von Elm et al., 2008). The Ethics Committee of Shanghai Seventh People’s Hospital approved the study (2024-7th-HIRB-098), and it is registered with the Chinese Clinical Trial Registry (ChiCTR2400092793).

### 2.2 Sample size calculation

The sample size for this study was determined through a priori power analysis performed using G*Power software (version 3.1.9.7) (Faul et al., 2007). Based on previous research, the study employed an Analysis of Variance (ANOVA) with repeated measures in the calculations. We expected an effect size of 0.40, an alpha error of 0.05 and a power level (1–β) of 0.20. The analysis indicated that at least 36 participants would be necessary to achieve an adequate cohort size for the study. Taking into account the participant dropouts rate 10%, the final sample size for this study was set at 40 individuals, with 20 participants allocated to each group. This sample size is considered adequate to generate reliable and valid outcomes for the research.

### 2.3 Participants

The study will be conducted in the Department of Rehabilitation at Shanghai Seventh People’s Hospital from August to September 2025. KOA patients will be recruited from the hospital’s outpatient clinics or nearby community health centers through posters displayed by doctors or therapists. Participants who express their interest in the study will be evaluated for eligibility to determine whether they meet the inclusion criteria for this research. At the same time, researchers will obtain consent in accordance with the principle of informed consent to confirm the voluntariness of the participants. The inclusion criteria: (1) age from 50 to 75 years; (2) KOA diagnosis following the American College of Rheumatology’s clinical criteria; (3) clinical and radiological signs in the right knee only; (4) Kellgren-Lawrence grade II or III; (4) chronic pain (present for no less than 3 months) exceeds 30 out of 100. The exclusion criteria: (1) a history of severe knee injury or rheumatic disorders; (2) a history of cerebrovascular disease, mental illness or brain metal implantation; (3) drug/alcohol abuse; (4) cognitive impairment or failure to cooperate. HC will be recruited from communities around the hospital via word-of-mouth and advertisements, who have no history of diseases affecting joints and brain. All individuals need to have a right lower limb dominance, determined by their kicking preference (Willems and Ponte, 2012). Before the study is carried out, all subjects are required to complete a written informed consent form in accordance with ethical review requirements.

### 2.4 Procedure

Before the experiment, the demographic data of all subjects will be first collected. We then explained the experimental process, test actions, and precautions to the participants. Adequate time will be provided for them to acclimate to the experimental process, ensuring a comprehensive understanding of the experimental requirements. Subsequently, fNIRS equipment and sEMG sensors will be affixed to each participant.

**FIGURE 2:**
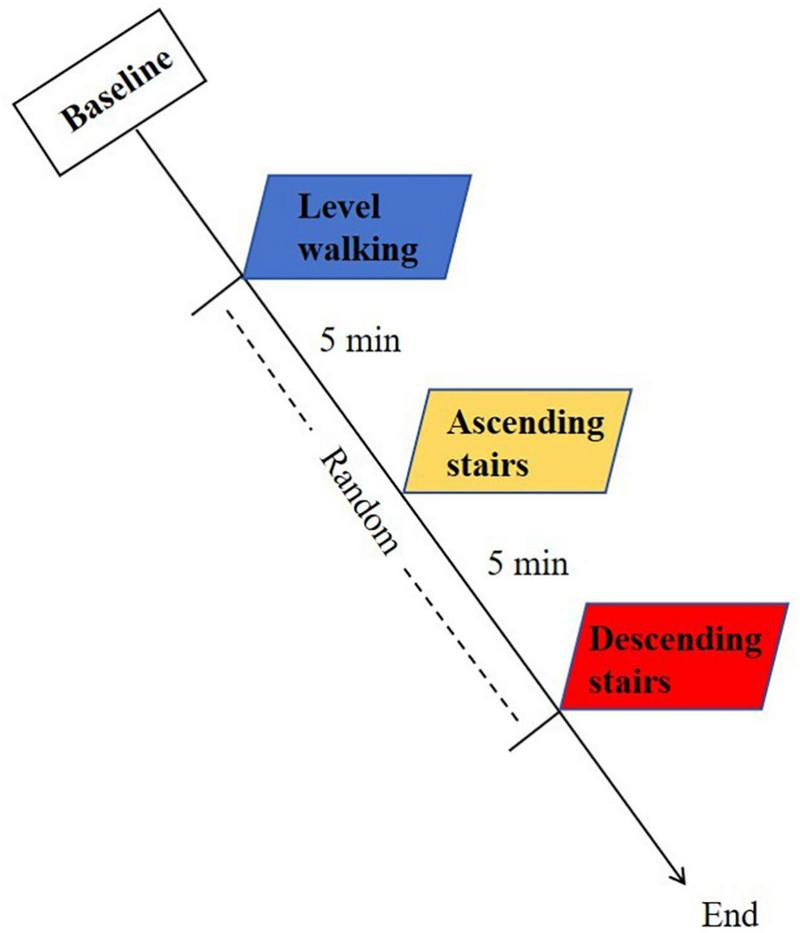
Schematic overview of the experimental protocol.

The experimental protocol consists of three distinct motor tasks: level walking, ascending stairs, and descending stairs. Participants will perform three tasks in a random order, with each task being repeated three times. There will be a 5 min interval between tests to allow participants to rest adequately and ensure that the fNIRS spectral signals of each test return to the baseline. Tests will be conducted on the KOA patients first, followed by the healthy controls. During the level walking, the patients will be instructed to walk at a normal, comfortable self-selected speed (SSS) for 30 s each time, with SSS defined as the average velocity from three timed baseline walks. The stair program evaluation will use standardized stairs under the same SSS conditions, maintaining consistent handrail non-use protocol across groups. The staircase consisted of 10 steps, with each step having a height of 12 cm, conforming to the standard specification for steps in China. To mitigate speed-related confounding effects, healthy controls will be pace-matched to the patient cohort’s average speed under the same conditions. A schematic overview of the experimental protocol is depicted in Figure 2. During the test, if the participant experiences any discomfort (e.g., dizziness, or falls), the testing will be immediately stopped and undergo medical evaluation of whether to continue.

### 2.5 fNIRS data acquisition and preprocessing

A portable multi-channel wireless fNIRS device (Brite24, Artinis Medical Systems, Netherlands) will be employed to capture cerebral fNIRS signals to evaluate alterations in brain activity patterns during movement. Following the international 10–20 system for electrode placement, the Cz electrode location of participants is identified using three-dimensional digitizer (Patriot, Polhemus): the intersection of the sagittal plane (connecting the nasion and inion) and the coronal plane (linking bilateral preauricular points) defines the standardized positioning of the Cz electrode. This will allow channel positions to be mapped to the Montreal Neurological Institute (MNI) coordinate system, enabling precise spatial normalization across participants. Subsequently, we will measure the head circumference of each participant to select a suitable size head cap equipped with a fNIRS probe. The elastic cap with adjustable sliding mounts allowed dynamic adaptation of source-detector spacing (25–35 mm) based on real-time scalp coupling verification via optical intensity monitoring. This hybrid spatial referencing strategy adapts to changes in scalp curvature and hair density, ensuring optimal light penetration and signal-to-noise ratio.

**FIGURE 3:**
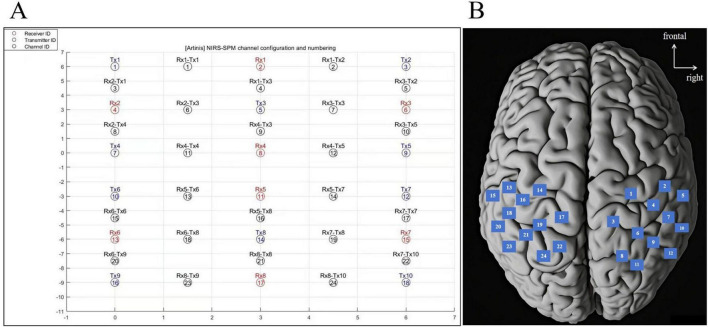
**(A)** Near-infrared spectroscopy-statistical parametric mapping toolbox (NIRS-SPM) channel configuration and numbering. **(B)** Schematic representation of fNIRS channel. fNIRS, functional near-infrared spectroscopy.

This system enabled the measurement of oxygenated hemoglobin (HbO) and deoxygenated hemoglobin (HbR) (Ferrari and Quaresima, 2012). The system emits near-infrared light at 670 nm and 850 nm wavelengths with a 10 Hz sampling frequency. Each sensor within the apparatus comprises a light-emitting diode paired with a detector photodiode. In total, the configuration includes 10 light sources and eight detectors, resulting in the formation of 24 channels. As shown in Figure 3. The three-dimensional digital recording of fNIRS channel positions mainly covers six specific brain regions of interest (ROIs) in both hemispheres: primary somatosensory cortex (S1), primary motor cortex (M1), and somatosensory association cortex (SAC). These ROIs focused in this study play an important role in sensory processing and motor execution in the central nervous system (Anders et al., 2004; Bhattacharjee et al., 2020; Sansare et al., 2024). In addition, our previous studies have found that these regions are associated with changes in KOA (Yang et al., 2024).

This study will use the near-infrared spectroscopy-statistical parametric mapping toolbox (NIRS-SPM) implemented in MATLAB to process raw light intensity data obtained from Oxysoft software. The hmrIntensity2OD function is used to convert the data to optical density after eliminating low-quality channels. Head motion artifacts are mitigated using moving standard deviation, spline interpolation, and wavelet artifact correction methods (Guan et al., 2024). The corrected signal is filtered with a bandpass filter to remove physiological noise. The optical density data is transformed into relative changes in HbO and HbR through the modified Beer-Lambert law. Because studies have demonstrated that the HbO concentration possesses a high signal-to-noise ratio, being the most sensitive indicator of local cerebral hemodynamic responses (Gagnon et al., 2012; Ohshima et al., 2023). Moreover, over time, it exhibits higher repeatability and stability (Strangman et al., 2002; Plichta et al., 2006). Therefore, HbO will be chosen as the follow-up analysis in this study. After data preprocessing, we will use the general linear model (GLM) in spectroscopy-statistical parametric mapping (SPM) to model the changes in the concentration of HbO, obtaining the regression coefficient β value for each task condition (Wei et al., 2024). The β value reflects the amplitude of the task-induced blood oxygen signal change and can be used as a quantitative indicator of the neural activation intensity in the corresponding channel region. The design matrix in the model incorporated block-based conditions (boxcar function) corresponding to the three motor tasks, which are convolved with the hemodynamic response function (HRF) to account for the temporal delay inherent in neural activity. Group-level analysis extracts the HbO concentration changes and β values in predefined brain ROIs for subsequent statistics.

### 2.6 sEMG data acquisition and preprocessing

A multi-channel wireless sEMG system (Ultium EMG, Noraxon, United States) will be employed to measure the activity of lower limb muscles during movement. This can reflect muscle activation and functional status. sEMG sensors are placed on the skin surface of target muscles to record the electrical signals generated during muscle contractions. Including a total of seven target muscles of right lower limb as follows: rectus femoris (RF), vastus medialis (VM), vastus lateralis (VL), biceps femoris (BF), tibialis anterior (TA), medial head of gastrocnemius (GM), and lateral head of gastrocnemius (GL). The sEMG sensor is placed at the most prominent part of the muscle belly, ensuring that the sensor positions did not interfere with normal movement. In addition, the sensor is wrapped with a skin membrane to prevent the sensor from loosening or falling during movement.

In this study, Script will be written using Matlab2019b version software to complete the batch processing of surface EMG data. Firstly, the EMG data will be filtered to eliminate various noises in the signal. Then, the DC bias is removed by means of deaveraging, followed by full-wave rectification of the pre-processed signal. The Hilbert transform is used to extract the signal envelope. To reduce individual differences, the Maximum Voluntary Contraction (MVC) will be used to normalize the EMG signals of each muscle (Chowdhury et al., 2013). The indices selected for this study included root mean square (RMS) and median frequency (MF) of frequency-domain features derived from fast Fourier transform.

### 2.7 Clinical scales

In the present study, the Visual Analog Scale (VAS) for pain is employed to assess clinical pain. Specifically, it is a self - reported means for patients to estimate their perception of knee pain on a scale ranging from 0 to 10 (He et al., 2022). KOA symptoms are assessed using the Western Ontario and McMaster Universities Osteoarthritis Index (WOMAC), a specific index for osteoarthritis that ranges from 0 to 96. It is derived from the combined quantitative assessments of knee pain, stiffness, and function (Gandek, 2014). VAS and WOMAC will be assessed before exercise.

### 2.8 Statistical analysis

The statistical analysis of this study covers clinical indicators, muscle and brain activation. Statistical analysis will be conducted on clinical indicators and muscle activation using SPSS 21.0 Software (IBM Corporation, New York, United States). Continuous variables with normal distribution will be presented as mean ± standard deviation, while non-normally distributed variables will be shown as median and quartiles. Categorical variables will be reported as frequency. The normality test will be performed using Shapiro-Wilk test. An independent *t*-test is applied when demographic data follow a normal distribution. Otherwise, the Mann–Whitney’s U test will be applied. The Chi-square test will be used to compare sex. Muscle activation in different groups (KOA and HC) and tasks (level walking, ascending stairs, and descending stairs) will be analyzed using mixed ANOVA or non-parametric tests (for non-normality). For brain activation analyses, a flexible factorial design (FFD) in SPM will be employed to construct a mixed-effects model including groups (inter-subject factors) and tasks (intra-subject factors) to evaluate group main effects, task main effects and their interactions. SPM will be thresholded with cluster-level false discovery rate (FDR) correction (*p* < 0.05). To explore the correlation between brain activation (HbO concentration and β value) and clinical indicators (VAS and WOMAC) in KOA patients, a multiple linear regression analysis will be performed to calculate the Pearson correlation coefficient and RMS value. Additionally, a non-linear regression prediction based on the random forest algorithm will be used to assess the importance of regression between various brain regions, muscle characteristics, and clinical indicators. All statistical tests will use a significance level of 0.05, with corrections for multiple comparisons made using the Bonferroni adjustment.

## 3 Discussion

This study systematically explores the differences in sensory-motor cortex activation patterns in patients with KOA under different walking environments. Specifically, it compares neural activation characteristics during level and stair walking. Using fNIRS, the research will reveal changes in brain activation in KOA patients across different exercise environments, providing new insights into the pathological mechanisms of KOA. In addition, this study will provide potential targets for clinical intervention in KOA by investigating the connection between changes in patient brain activation and clinical indicators.

The feasibility of this study is underpinned by the robust methodology and the use of advanced neuroimaging techniques. The fNIRS system, with its ability to measure hemodynamic changes in the brain, offers a non-invasive and portable means of assessing cortical activation (Menant et al., 2020; Huo et al., 2023). Unlike fMRI, which imposes strict motion restrictions and requires a shielded laboratory environment, fNIRS enables non-invasive monitoring of cortical hemodynamic responses in naturalistic settings, including free movement and social interactions (Pereira et al., 2023). Cao et al. (2025) used FNIRS to study the brain activation characteristics of anterior cruciate ligament reconstruction (ACLR) patients during a repetitive upstairs task. The research results indicate that ACLR patients exhibit significant negative activation in the ipsilateral pre-motor cortex, supplementary motor cortex, and primary somatosensory cortex, while this feature was not observed in healthy individuals. The author believes that interventions targeting these brain regions may represent a novel rehabilitation approach. Another study used fNIRS to collect brain activation from KOA patients and healthy people while walking under exercise, and found that compared with comfortable walking, KOA patients had a significant increase in activation of the prefrontal cortex during disturbed walking, and this increase was more significant in healthy elderly women (Bishnoi et al., 2024). fNIRS portability and tolerance to motion artifacts make it ideal for investigating dynamic processes in real-world scenarios (Zinos et al., 2024). In addition, this study combined with sEMG measuring muscle activity could comprehensively evaluate the motor and functional status in KOA patients (Wang et al., 2023; Huang et al., 2025). This provides a physiological basis for near-infrared brain functional imaging, revealing the complex interaction mechanisms between peripheral muscles and the central nervous system in motor control, and analyzing nervous system adaptation processes at the muscle level. Huang et al. (2025) used fNIRS and sEMG technology to study the complex relationship between brain activation and muscle activation in healthy subjects while performing lower-limb exercise tasks. The results found a significant correlation between brain and muscle activation, highlighting the complex interaction between central and peripheral nervous mechanisms in motor control. The authors believe that these findings are important for clarifying motor control mechanisms and designing effective rehabilitation strategies. Clinically, this study holds significant promise for improving the management of KOA. By identifying specific brain regions that show altered activation patterns, we can potentially develop targeted interventions that address both the physical and neurological aspects of the disease. This could lead to more personalized treatment plans, enhancing patient outcomes and quality of life. A preliminary study by Hyochol et al. using fNIRS to measure the effects of transcranial direct current stimulation on patients with KOA has shown that central intervention is feasible and effective for KOA patients (Ahn et al., 2020). This study, by exploring the relationship between the clinical symptoms of patients with KOA and the relevant brain regions, may also contribute to the development of early predictive markers for KOA onset, facilitating timely interventions and better disease management.

However, this study also has its limitations. The cross-sectional design means that we cannot establish causality between brain activation patterns and KOA progression. Additionally, uncontrolled speed differences may confound neuromuscular and cortical activation patterns despite standardized pacing. Finally, the study is limited to a specific age range and severity of KOA, which may not be representative of all patient populations. Future studies should adopt longitudinal design to track dynamic neural adaptations relative to disease progression. Integrating speed as a covariate into a multivariate model to analyze its confounding effects. Large-sample stratified design that includes a broader age range and varying severities of KOA could provide more comprehensive insights into the disease’s impact on brain and muscle function.

In summary, this study aims to provide a detailed understanding of the brain activation patterns in KOA patients during different walking tasks. The innovative use of fNIRS and sEMG, combined with a robust methodology, ensures the feasibility and clinical relevance of this research. While the study has its limitations, the findings have the potential to significantly advance our understanding of KOA and inform the development of more effective intervention strategies.
